# 

**DOI:** 10.1192/bjb.2017.18

**Published:** 2018-08

**Authors:** Lisa Conlan

**Affiliations:** Consultant Psychiatrist, South London and Maudsley National Health Service Foundation Trust; email: lisa.conlan@slam.nhs.uk

I am a big fan of short books and, at around 100 pages, this is a remarkably short book. The authors outline the debate between autonomy and paternalism, and also aim to demonstrate where philosophy can be useful to psychiatry and medicine. The book succeeds in both areas, although in places it could have benefited from further elaboration and exposition.

Part 1 summarises the arguments for personal autonomy, defined as ‘acting on one's own reasons, not on those of other people’. The authors outline why this is valued as a good in its own right, only to be interfered in with significant moral justification. Paternalism is shown generally to act to undermine individual autonomy (although it can be justified e.g. for public health). Medicine is defined ‘not as a science but an interpretive practice relying on clinical reasoning’. The patient–doctor consultation is rightly situated as the ‘central act of medicine’. The way people make decisions is considered: not as isolated units but in dialogue with their significant others.

The best section is on Miranda Fricker's concept of epistemic injustice. Fricker has highlighted two types of injustice: testimonial and hermeneutical. Testimonial injustice refers to a speaker being awarded less credibility owing to prejudice on the listener's part, e.g. a woman speaking in a male-dominated boardroom. Psychiatric patients are particularly susceptible to this *credibility deficit*. The authors cite Elyn Saks's account of attending the accident and emergency department with a brain haemorrhage. When doctors heard of her history of schizophrenia, they stopped diagnostic investigations and sent her home. Hermeneutic injustice occurs when an individual/group does not have access to, or is not represented within, the shared generation of meaning. Here the injustice is often to the subject who questions the credibility of their own testimony. An example is a depressed new mother trying to make sense of her feelings without any knowledge of postnatal depression.

Part 2 addresses specific problems: mental health legislation, do-not-resuscitate orders, and assisted dying. These are condensed and well-written, but there is nothing new for anyone working clinically in these specialties.

Overall, this is a clear and concise introductory work useful for anyone interested in this area.

